# Distribution of the Unionida (Bivalvia, Paleoheterodonta) from Argentina and its conservation in the Southern Neotropical Region

**DOI:** 10.1371/journal.pone.0203616

**Published:** 2018-09-11

**Authors:** Santiago Torres, Luciana Cao, Diego Eduardo Gutiérrez Gregoric, Micaela de Lucía, Francisco Brea, Gustavo Darrigran

**Affiliations:** 1 División Zoología Invertebrados, Museo de La Plata, Facultad de Ciencias Naturales y Museo, Universidad Nacional de La Plata (FCNyM-UNLP), La Plata, Argentina; 2 Centro de Investigación y Transferencia Santa Cruz, Argentina (Consejo Nacional de Investigaciones Científicas y Técnicas–CONICET- / Universidad Nacional de la Patagonia Austral -UNPA- / Universidad Tecnológica Nacional -UTN-), Río Gallegos, Argentina; 3 CONICET, La Plata, Argentina; 4 Comisión de Investigaciones Científicas de la Provincia de Buenos Aires (CIC), La Plata, Argentina; Australian Museum, AUSTRALIA

## Abstract

Bivalves are one of the most representative groups in the Phylum Mollusca, with over 1,100 freshwater species around the world except Antarctica. About 900 of these species belong to the Order Unionida Gray, 1854. In South America, the distributional range of the Unionida includes all countries in the region and extends as far south as the lakes and rivers of Argentinean-Chilean Patagonia. With the aim of generating distribution maps for the different genera of Unionida in Argentina, we consulted the databases of the nation’s main official malacological collections. The data were analyzed and georeferenced using the point-radius method. Spatial analyses were performed with the software Q-GIS 2.16.3 Nødebo using vector layers under the 2007 Argentinean Geodesic Positions reference system. A total 1,833 lots were analyzed, of which it was possible to georeference 1,503. The distribution of Unionida in Argentinean territory was analyzed according to political provinces, Surface Drainage Basins and the Argentinean Protected Areas. Species richness was analyzed using the surface drainage basins of Argentina. We generate distribution maps for each genus and discuss the species threat status and conservation degree in the region. Only six (18%) of the Unionida present in Argentina have been classified by the IUCN, four are *Least Concern* and two are *Data Deficient*. This pattern is also valid for all of South America. More than 95% of the distributional range of the Unionida has no protected area. Conservation management is necessary for the preservation of Unionida diversity in southern South America.

## Introduction

Bivalves are one of the most representative groups in Phylum Mollusca, with over 1,100 freshwater species around the world except in Antarctica [1]. About 900 of these species belong to the Order Unionida Gray, 1854. Over half of them are distributed in the Palearctic and Indotropical Regions, which constitute the world’s main regional hotspots [2], [3]. The Neotropical Region is the third most diverse for Unionida [3], [4], with 249 species distributed in eight families [1]. In South America, the distribution range of the Unionida includes all the countries in the region [5] and extends as far south as the lakes and rivers of the Argentinean-Chilean Patagonia [6], [7], [8], [9].

Unionidan species (naiads) are difficult to identify because the morphological features traditionally considered of taxonomic value are few and highly variable, creating problems both for identification and for the study of phylogenetic relations in the Neotropical Region [10], [11].

Pereira et al. [5] propose that the number of valid species for South America is 112, distributed among 15 genera. Brazil, Argentina and Uruguay are the countries with greatest species diversity. The Brazilian Amazon Basin, with over 40 recorded species, has the greatest richness, followed by the Paraná and Uruguay River Basins with 30 to 40 species, respectively. According to these authors, the unionidan families present in Argentina are Mycetopodidae and Hyriidae. Table 1 presents a checklist of the Unionida present in Argentina following the systematics of Pereira et al. [5], their presence in the Argentinean provinces according to Rumi et al. [9] and the revision of the unionidan database (S1 Appendix). Whereas the Mycetopodidae are exclusively a Neotropical family, the Hyriidae has a wide distribution, including South America, Australia, New Zealand and New Guinea [4]. Despite the difficulty of identification at a specific level, several South American malacologists have provided descriptions based on conchological characters, internal anatomy of soft tissues and descriptions of larval stage [6], [12–22]. For a better understanding of the Order, a synthesis of the groups of Unionida present in Argentina is provided.

**Table 1:** Checklist of unionida and presence in Argentinean provinces. Jujuy (JU), Salta (SA), Tucumán (TC), Formosa (FM), Chaco (CO), Misiones (MN), Corrientes (CN), Entre Ríos (ER), San Luis (SL), Santiago del Estero (SE), Mendoza (MZ), Córdoba (CB), Santa Fe (SF), Buenos Aires (BA), Neuquén (NQ), Río Negro (RN), Chubut (CH), Catamarca (CT). Numbers indicates quantity of lots.

	JU	SA	TC	FM	CO	MN	CN	ER	SL	SE	MZ	CB	SF	BA	NQ	RN	CH	CT
Unionida																		
Mycetopodidae																		
*Anodontites* Bruguiére, 1792	8	8	4	11	18	39	75	152	1	4		4	93	233	1	11		2
*A*. *(A*.*) elongatus* (Swainson, 1823)														X				
*A*. *(A*.*) lucidus* (Orbigny, 1835)							X	X					X					
*A*. *(A*.*) patagonicus* (Lamarck, 1819)	X	X	X	X	X	X	X	X	X	X		X	X	X				X
*A*. *(A*.*) puelchanus* (Orbigny, 1835)															X	X		
*A*. *(A*.*) soleniformis* (Orbigny, 1835)						X	X	X					X					
*A*. *(A*.*) tenebricosus* (Lea, 1834)						X	X	X					X	X				
*A*. *(A*.*) trapesialis* (Lamarck, 1819)	X	X	X	X	X	X	X	X	X	X		X	X	X				X
*A*. *(A*.*) trapezeus* (Spix, 1827)						X	X	X					X	X				
*A*. *(Lamproscapha) ensiformis* (Spix, 1827)				X	X	X							X	X				
*Mycetopoda* d’Orbigny, 1835				2	1		6	17					23	54				
*M*. *legumen* (Martens, 1888)				X			X	X					X	X				
*M*. *siliquosa* (Spix, 1827)					X		X	X					X	X				
*M*. *soleniformis* Orbigny, 1835							X	X					X	X				
*Monocondylaea* Orbigny, 1835						6	10	17					14	3				
*M*. *corrientesensis* (Orbigny, 1835)						X	X	X					X	X				
*M*. *minuana* (Orbigny, 1835)						X	X	X						X				
*M*. *paraguayana* (Orbigny, 1835)						X	X	X					X	X				
*M*. *parchappii* (Orbigny, 1835)					X		X											
*Fossula* Lea, 1870							3											
*F*. *fossiculifera* (Orbigny, 1835)						X	X											
*Leila* Gray, 1840		2		2				4					7	10				
*L*. *blainvilliana* (Lea, 1834)		X		X				X					X	X				
Hyriidae																		
*Diplodon* Spix, 1827		2		11	12	44	75	148					98	309	42	44	4	
*D*.*(D*.*) chilensis* (Gray, 1828)											X			X	X	X	X	
*D*. *(D*.*) delodontus* (Lamarck, 1819)								X					X	X				
*D*. *(D*.*) parallelopipedon* (Lea, 1834)				X	X	X	X	X					X	X				
*D*. *(D*.*) parodizi* Bonetto, 1960				X	X			X					X	X				
*D*. *(D*.*) rhuacoicus* (Orbigny, 1835)								X						X				
*D*. *(D*.*) wymanii* (Lea, 1860)								X										
*D*. *(Rhipidodonta) burroughianus* (Lea, 1834)							X	X					X	X				
*D*. *(R*.*) charruanus* (Orbigny, 1835)				X			X	X					X	X				
*D*. *(R*.*) hyaleus* (Orbigny, 1835)				X	X	X	X	X					X	X				
*D*. *(R*.*) peraeformis* (Lea, 1860)							X											
*D*. *(R*.*) variabilis* (Maton, 1811)							X	X					X	X				
*D*. *paranensis*				X	X		X	X						X				
*Castalia* Lamarck, 1819		2		6	1	4	31	28					49	59				
*Castalia inflata* Orbigny, 1835		X		X		X	X	X					X	X				
*C*. *psammoica* (Orbigny, 1835)				X		X	X	X					X	X				
*Total of species*	2	5	2	12	9	14	23	24	2	2	1	2	22	25	2	2	1	2
*Total of lots*	8	14	4	32	32	93	200	366	1	4	0	4	284	668	43	55	4	2

For the systematic Pereira et al. [5] was followed. Checklist in Argentinean provinces was made according with Rumi et al. [9] and the revision of the unionidan database (S1 Appendix).

### Mycetopodidae

The Mycetopodidae is represented by 19 species, distributed in five genera [5]: *Anodontites*, *Mycetopoda*, *Monocondylaea*, *Fossula* and *Leila*. The genus *Anodontites* is the most widely distributed within the family and includes the highest number of species (10) recorded in Argentina (Table 1).

### Hyriidae

The family Hyriidae is represented by two genera (*Diplodon* and *Castalia*] with 14 species for Argentina (Table 1). The genus *Diplodon* is the most widely distributed naiad not only in Argentina, but also in all South America, with a distribution that ranges from Venezuela to Patagonia [5].

Despite its wide distribution in almost every continent, the rate of extinction of the Unionida is very high compared to other groups like vertebrates [23], [24], [25]. The 2015 IUCN Red List of Threatened Species classified 224 of the 551 worldwide freshwater mussel species as Near Threatened or Threatened. Factors like habitat loss and fragmentation, overexploitation, pollution, loss of host fishes, invasive species, water abstraction and climate change among others are causing the populations of Unionida to decline globally [23], [26], [27].

In South America, mainly in the mid-twentieth century, industrial-scale nacre extraction from naiads had a strong negative impact on natural populations. However, it also enhanced pioneering research in Argentina and the region, where experiments were being conducted on repopulation and sustainable species management [17], [28]. When nacre was replaced by plastic, the use of the resource declined, leading to a slower rate of decrease in mussel populations caused by extraction activities [10]. Nowadays a few sites for naiad population exploitation remain in the Brazilian Amazon [29]. Nevertheless, pollution caused by the plastic industry, among others, has again reduced Unionida populations in recent decades [30].

A new threat appeared in the region in the mid-1980s and early 1990s –the introduction of invasive bivalve species such as *Corbicula fluminea* (Müller, 1774) and *Limnoperna fortunei* (Dunker, 1857)​​ [31], [32]. *Limnoperna fortunei* has an aggressive impact on the environment because it is an ecosystem engineer [32]. These bioinvasions have changed the structure of communities in impacted freshwater bodies, probably causing further reduction of Unionidan species abundance [30], [33].

Although taxonomical and biological studies of Unionida in Argentina increased during the 1960s and 70s [6], [12], [14], [15], [17], [18], [34], [35], [36], [37] they were discontinued in the late twentieth and early twenty-first centuries, leading to deficient knowledge on current populations. The species *Diplodon* (*Diplodon*) chilensis (Gray, 1828) is an exception, as research has been conducted into the physiology and toxicology of its populations in Patagonia [38], [39].

According to Klunzinger et al. [40], precise delimitation of the geographical range of a species is important for conservation planning and biogeographic studies. Additionally, this type of research provides current environmental data through sampling activities and historical environmental data through biological collections.

The aim of this study is to georeference the Unionida lots contained in Argentina’s main malacological collections, to generate maps of distributional range at genus level and compare them to other distributions included in the available literature of the Unionida. The results of this study will shed light for further research, to estimate the degree of threat for the unionidan species [41] and develop plans for creating potential conservation priority areas.

## Materials and methods

**Fig 1:**
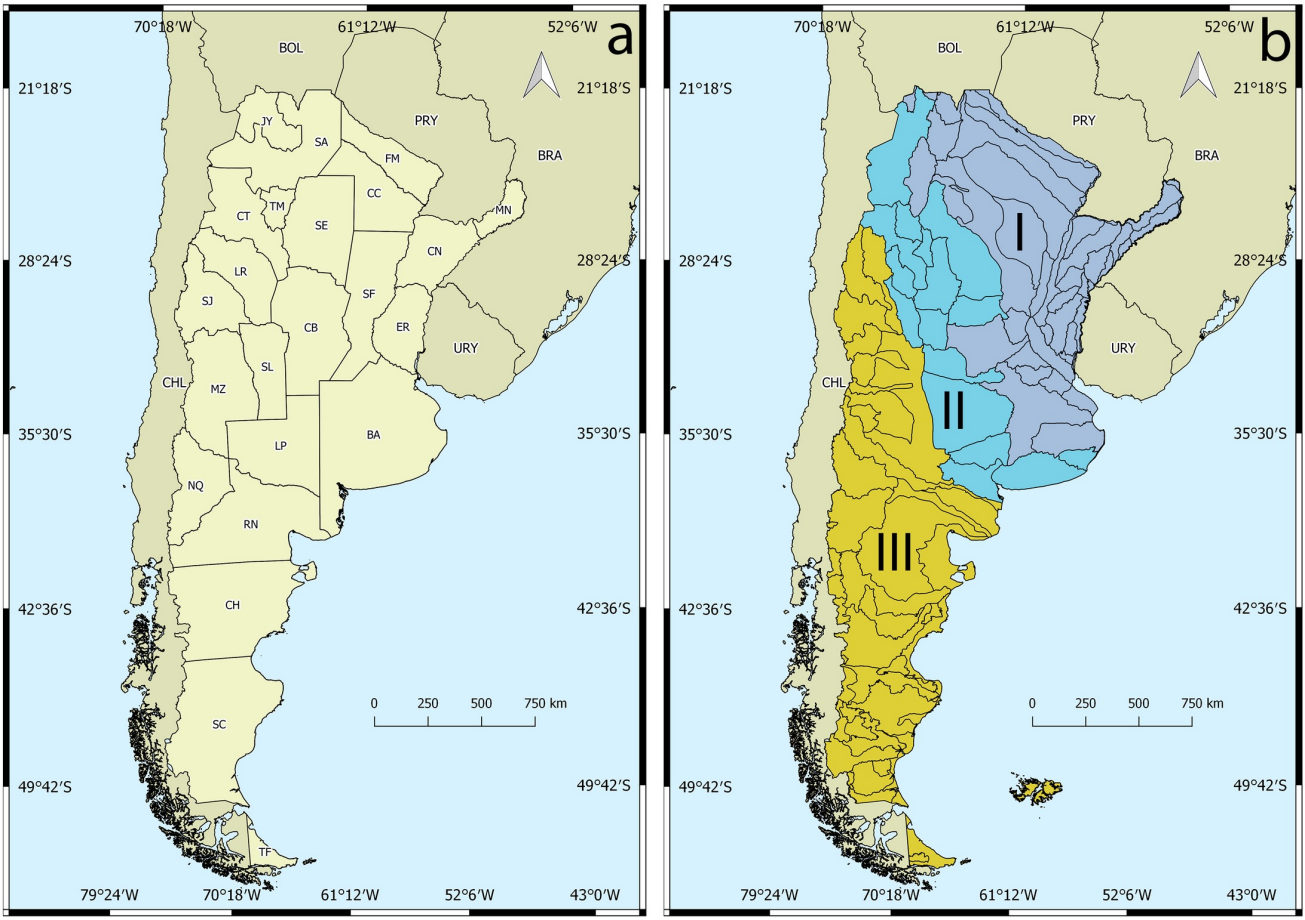
a) Political division of Argentina: Jujuy (JU), Salta (SA), Tucumán (TC), Formosa (FM), Chaco (CO), Misiones (MN), Corrientes (CN), Entre Ríos (ER), Catamarca (CT), San Juan (SJ), La Rioja (LR), San Luis (SL), Santiago del Estero (SE), Mendoza (MZ), Córdoba (CB), Santa Fe (SF), La Pampa (LP), Buenos Aires (BA), Neuquén (NQ), Río Negro (RN), Chubut (CH), Santa Cruz (SC), Tierra del Fuego, Antártida e Islas del Atlántico Sur (TF). b) Surface Drainage Basins of Argentina [40], [41] in South American Hydrographic regions [43]. **I**: The Plata basin, **II**: Border strip of the Brasilica (North) and the Chilean-Patagonian (South West) Subregion and **III**: Chilean-Patagonian Subregion of the Atlantic Versant.

The databases of Argentina’s main malacological collections containing unionidan specimens were reviewed: Museo de La Plata (MLP), Museo de Ciencias Naturales “Bernardino Rivadavia” (MACN), Fundación Miguel Lillo (FML) and Museo de Santa Fe (MSF). For distributional analysis, all the information of the collections was used at a generic level. Data were georeferenced using the point radius method following the protocols set forth by Wieckzorec et al. [42]. The South American Hydrographic Regions proposed by Bonetto [43] were followed (Fig 1). The Surface Drainage Basins of Argentina were provided by the National Hydrological Information System of the National Sub-secretariat of Hydrological Resources of Argentina (SsRH) (Fig 1A) [44], [45]. A complete description of the Surface Drainage Basins can be found in Giraut et al. [44] and SsRH [45]. The Argentinean Protected Areas (APA) used in this work represent both National and Provincial jurisdictions [46], [47]. The maps of APA used in the present work were provided by Sistema Federal de Áreas Protegidas (SiFAP) [46]. For all spatial analyses, all vector layers were used under the Argentinean Geodesic Positions reference system with a Gauss-Krüger flat projection that divides the world into bands [48]. Species richness was analyzed using the surface drainage basins of Argentina and the presence of species of naiads in those basins following Rumi et al [49]. All the maps presented in this work were generated by the free-software Q-GIS 2.16.3 Nødebo.

## Results

### Distribution of unionida in surface drainage basins

**Fig 2:**
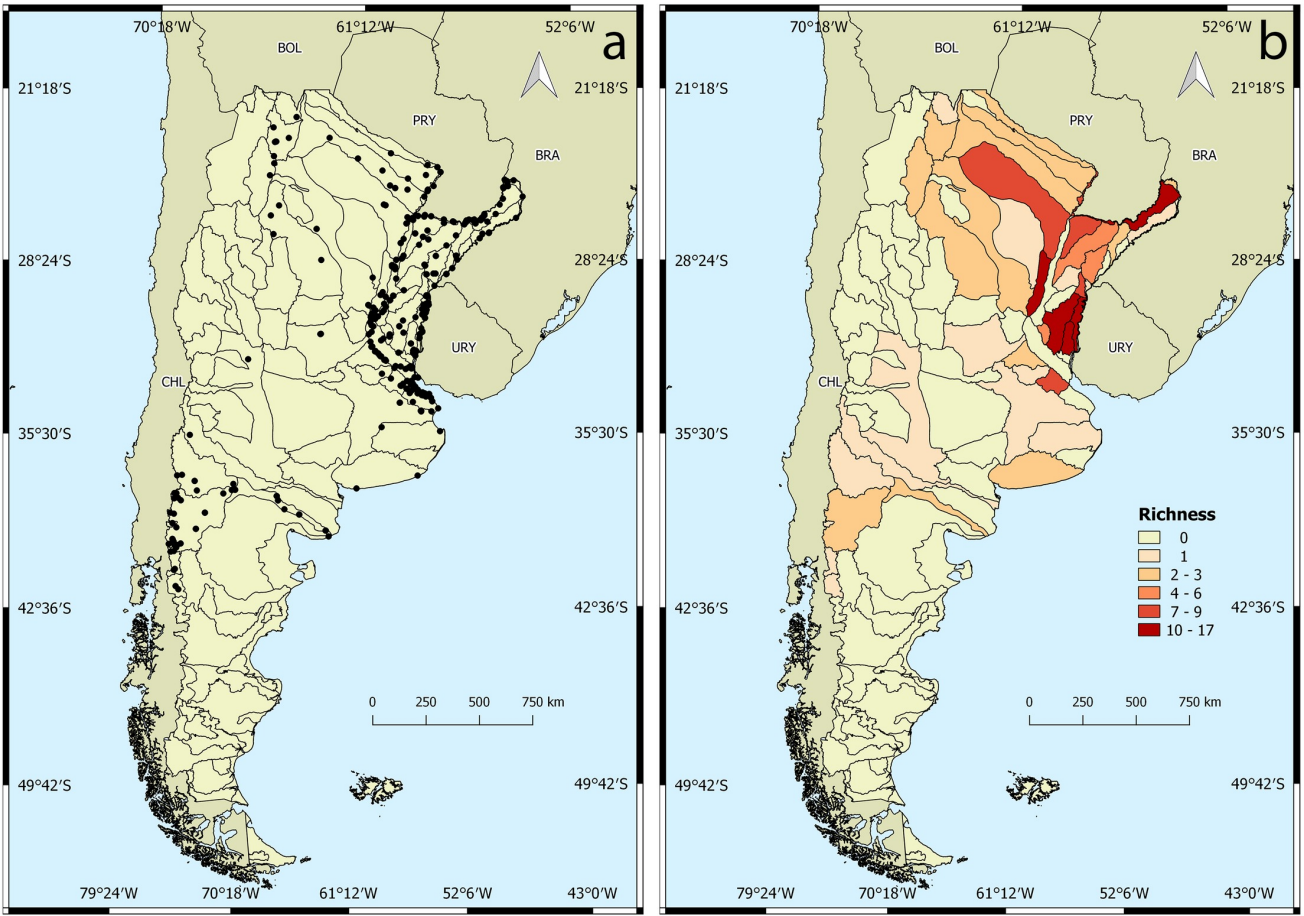
Spatial analysis of unionida in Argentina. a) Georeferenced lots in surface drainage basins. b) Unionida richness.

A total of 1,833 lots was analyzed (58% of the lots were from MACN collections, 41% from MLP and 1% from FML and MSF). Of these, 1,503 lots were georeferenced (Fig 2B). The remaining 330 lots could not be georeferenced because of lack of information about the collection localities, including lots from the FML and MSF. A list with basic information of the lots used in this work is presented in S1 Appendix. Eighteen of the 23 Argentinean political provinces have at least one naiad species (Table 1). Naiad distribution ranges from northern Argentina (23°9'28"S, 64°19'47"W) (Jujuy Province) to the Patagonian lakes in the south (42°58'35"S, 71°31'52"W) (Chubut Province).

The Plata Basin was the hydrographic system with the highest richness, with species predominantly in the Paraná and Uruguay rivers and their tributaries (Fig 2C). Patagonia presented only two genera (*Diplodon* and *Anodontites*) with one species each, *D*. *(D*.*) chilensis* and *A*. *(A*.*) puelchanus*.

#### Mycetopodidae

**Fig 3:**
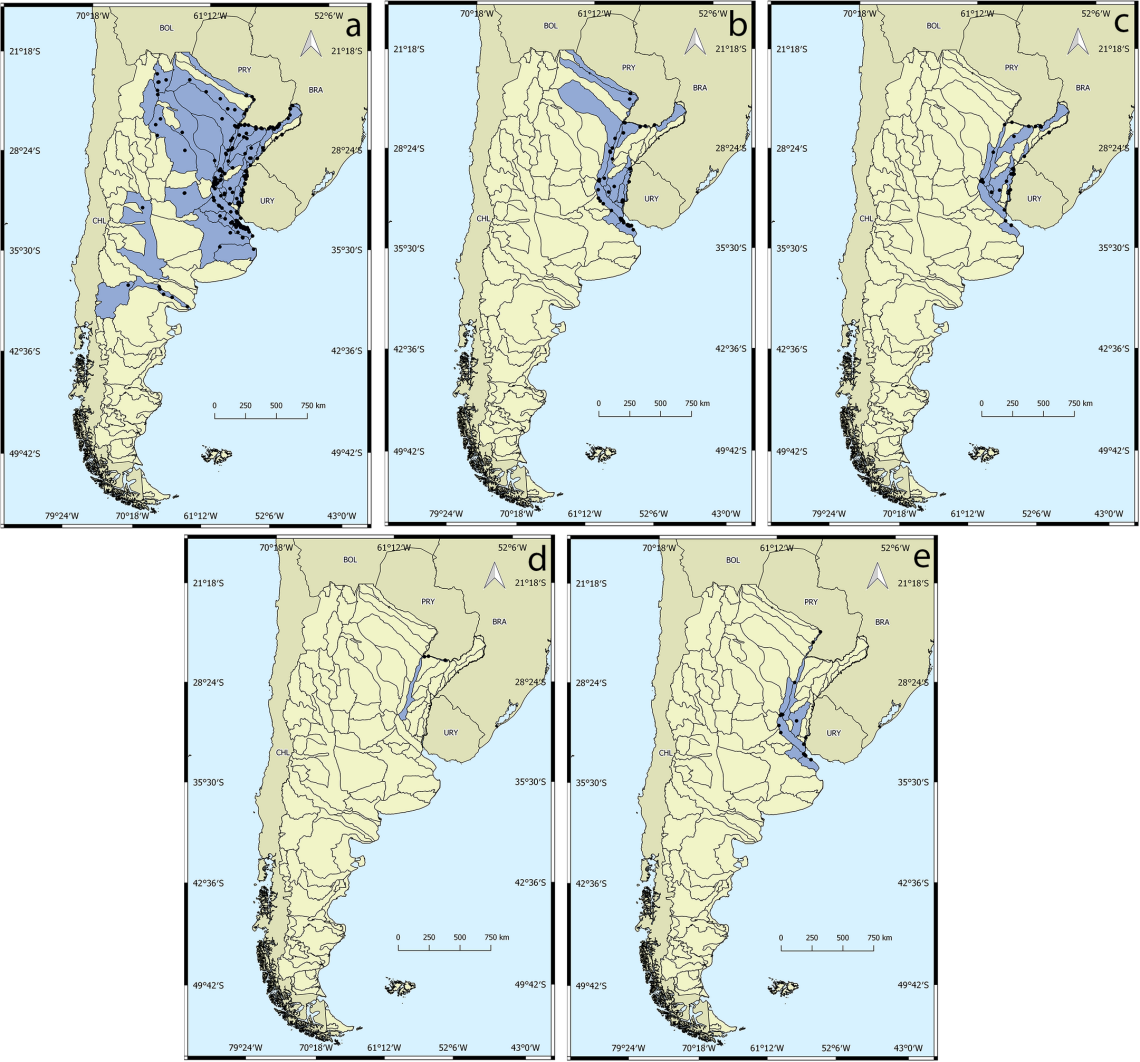
Distribution of genera in the family Mycetopodidae. a) *Anodontites*, b) *Mycetopoda*, c) *Monocondylaea*, d) *Fossula*, e) *Leila*. Black dots indicate georeferenced lots for each genus. Blue areas show surface drainage basins with presence of the genera.

Its distribution ranges from northern Argentina (MACN: 20852) to Patagonia (MACN: 20240), mainly in the Río de la Plata, Paraná and Uruguay rivers (Fig 3A–3E).

### Genus *Anodontites*

The genus *Anodontites* is the second most diverse in the region. *Anodontites* species are present in 38 Argentinean basins (Fig 3A). Their distribution ranges from the Pucará de Tilcara in the San Francisco River Basin (MACN 20852) to the Río Negro Basin (MACN: 20240), located in the Patagonian region. The highest number of species is mainly concentrated in The Plata Basin (seven species). The most widely distributed species is *A*. *trapesialis* (Lamarck, 1819), which has been recorded from the San Francisco River Basin from northern Argentina (MLP 4522) to the Buenos Aires Province (MLP 13068).

### Genus *Mycetopoda*

Three species of *Mycetopoda* are cited for Argentina, *M*. *legumen (Martens*, *1888)*, *M*. *siliquosa* (Spix, 1827) and *M*. *soleniformis* Orbigny, 1835, which are mainly present in The Plata Basin. *Mycetopoda* lots were recorded in 18 basins, mainly on the Paraná and Uruguay rivers (Fig 3B).

### Genus *Monocondylaea*

The genus *Monocondylaea* has records in 12 basins on the Paraná and Uruguay rivers and their tributaries, with some records in the Río de la Plata (MLP 13195; MLP 5809) (Fig 3C). Four species are cited for Argentina (Table 1). *Monocondylaea corrientensis* (Orbigny, 1835) and *M*. *paraguayana* (Orbigny, 1835) are the species with greatest distribution range with records in the provinces of Misiones, Corrientes, Entre Ríos and Buenos Aires.

### Genus *Fossula*

This genus is monotypic, *Fossula fossiculifera* (d'Orbigny, 1835). Three lots were georeferenced for *F*. *fossiculifera*, all on the Paraná River: (MLP 5844); (MLP 1775–1); (MLP 5750) (Fig 3D).

### Genus *Leila*

Almost all the georeferenced records of *Leila* are related to the Paraná and Uruguay rivers and their tributaries (Fig 3E) and some records were located in the Río de la Plata River. Only one species in Argentina, *Leila blainvilliana* (Lea, 1834) (MACN 6287, 6342, 7782).

#### Hyriidae

**Fig 4:**
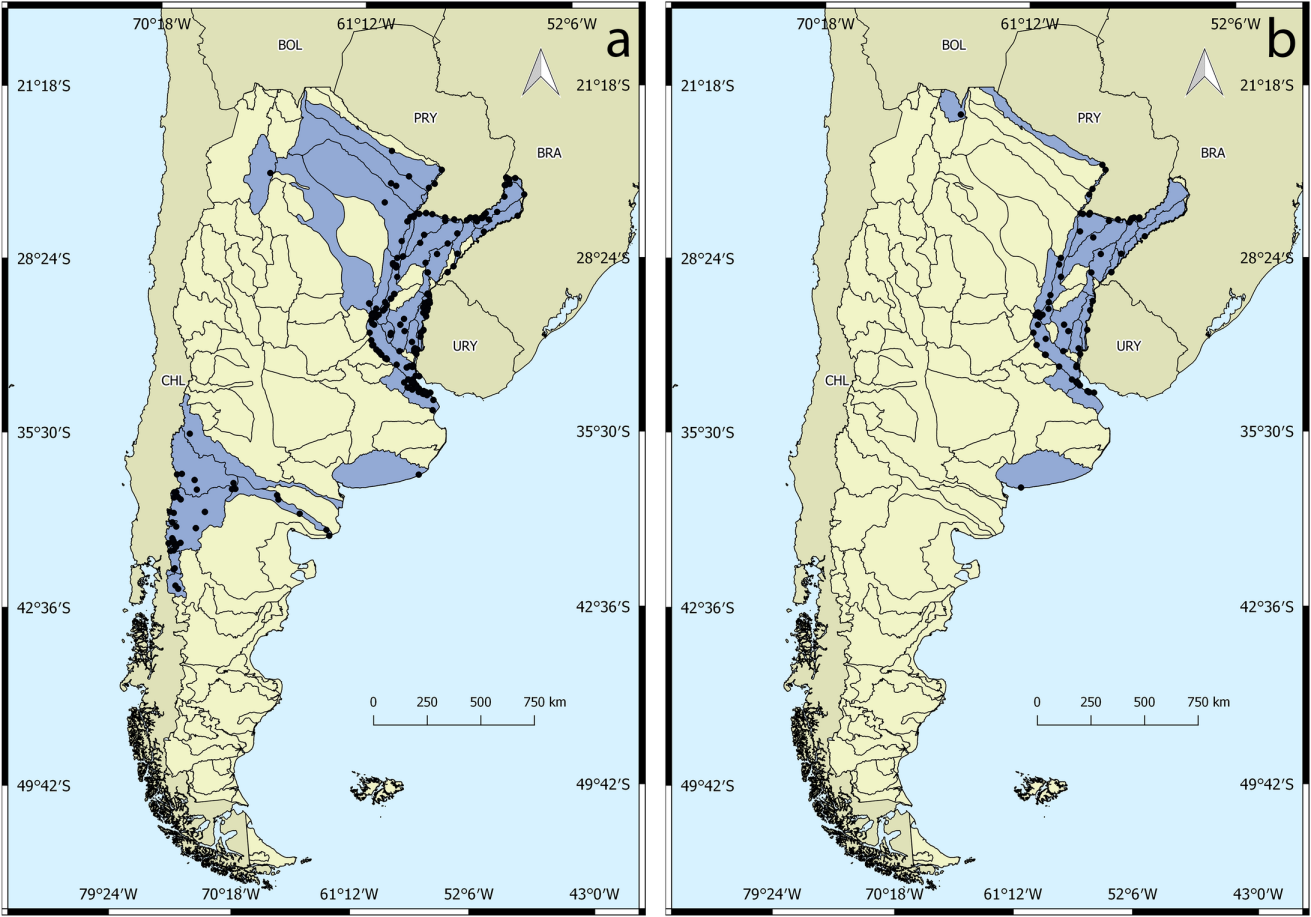
Distribution of genera of the family Hyriidae. a) *Diplodon*, b) *Castalia*. Black dots indicate georeferenced lots for each genus. Blue areas show surface drainage basins with presence of the genera.

The distribution of the Hyriidae family ranges from the upper basin of the Bermejo River in Salta Province (MLP 6377; 6376) to Lake Futalaufquen in Patagonia (MACN 25894). In total, 806 lots were georeferenced, with records in 56 drainage basins (Fig 4A and 4B).

### Genus *Diplodon*

The genus *Diplodon* is the most widely distributed, with records in most basins where the family is present, mainly the basins of the Paraná and Uruguay rivers and their tributaries. A total of 649 lots of the genus *Diplodon* were georeferenced, with 12 species distributed over 33 drainage basins, mostly belonging to The Plata Basin (Fig 4B). *Diplodon (D) chilensis* is the only species of Hyriidae present in Patagonia, very common in lakes and rivers in the region.

### Genus *Castalia*

Two species were cited for Argentina, *Castalia inflata* Orbigny, 1835 and *C*. *psammoica* (Orbigny, 1835), with records in 23 drainage basins, mainly on the Paraná and Uruguay rivers and their tributaries (Fig 4B). Their distribution ranges from the upper basin of the Bermejo River (MLP 6377; 6376) to southern Buenos Aires Province (MLP 1413).

### Distribution of unionida in the Argentinean Protected Areas (APA)

**Fig 5:**
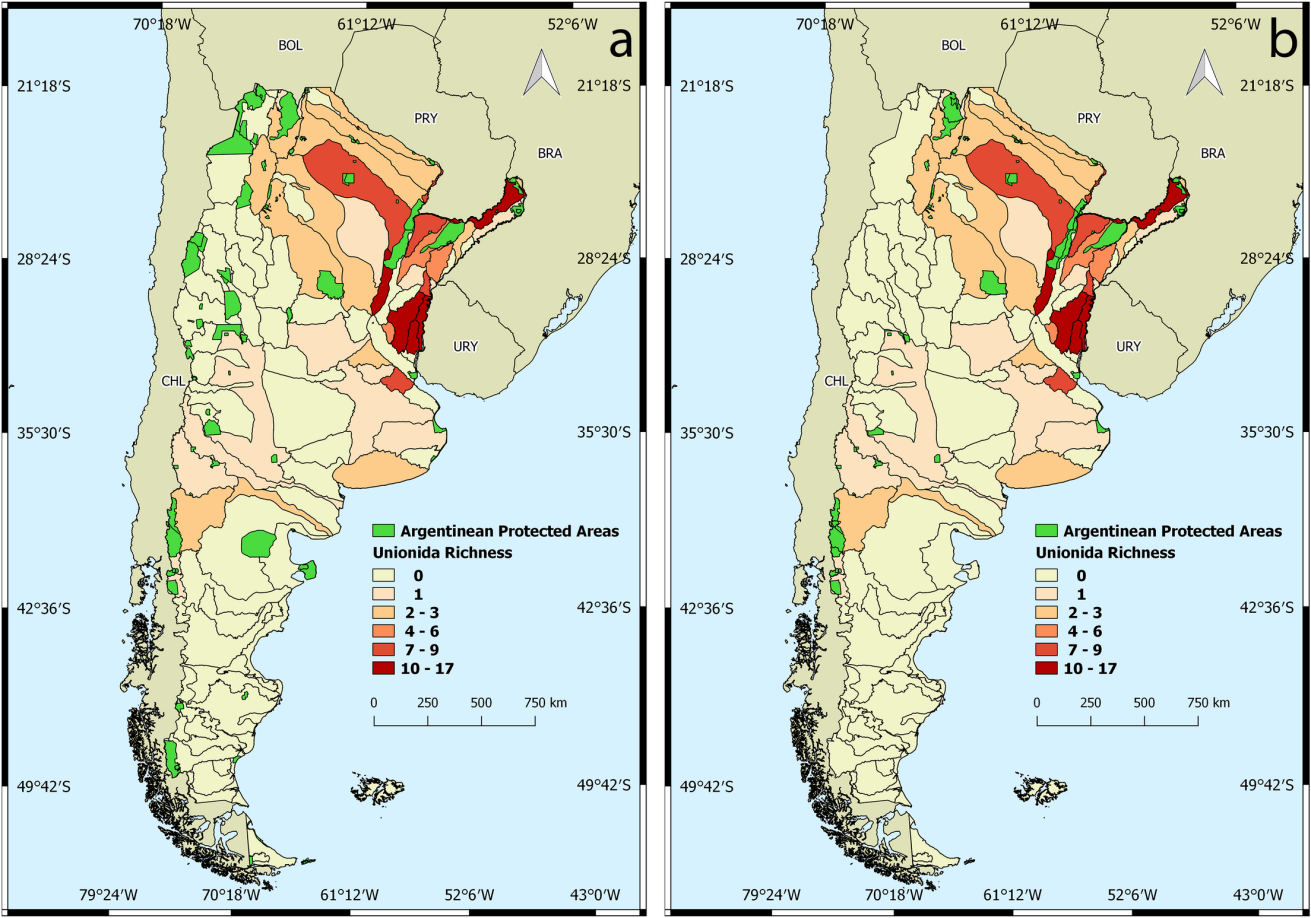
Spatial analysis between naiad presence in the Argentinean Surface Drainage Basins and the Argentinean protected areas (APA). a) APA and Unionida richness. b) APA within Unionida richness distribution.

The APA cover less than 15% of the national territory (Fig 5A), protecting a small but significant part of the ecoregions in southern South America. A total of 51 basins have at least one APA. The results of the distribution of naiads in the APA shows that only eighteen of the APA have at least one record of Unionida. Nine correspond to a National Park jurisdiction: Copo, Pilcomayo, Iguazú, Nahuel Huapi, Lanín, Los Alerces, Lago Puelo and El Palmar; two correspond to a Provincial Park jurisdiction: Salto Encantado (Misiones), Iberá (Corrientes); three correspond to a biosphere reserve: Yungas, Yabotí and Delta del Paraná; four correspond to a Ramsar site: Jaaukanigas, Lagunas and Esteros del Iberá, Chaco wetlands, Pilcomayo river. Only twelve APA have four or more species of naiads cited in the area. This means that less than 10% of the protected areas on the national territory has a significant richness of Unionida. The distributional range of the unionidan presented in this work indicates that more than 95% of this area is not in a protected habitat (Table 2). Most of the basins with four or more species of naiads are part of The Plata Basin, which is also the hydrographic system with the lowest number of protected areas in Argentina (Fig 5B).

**Table 2:** Area analysis of unionida distribution.

	SDB^a^	SDB/Unionida^b^	APA^c^	APA/Unionida^d^
Area (Km^2^)	2,791,810	1,119,852	350,000	116,916

^a^ Surface Drainage Basins

^b^ Surface Drainage Basins with presence of Unionida

^c^ Argentinean Protected Areas

^d^ Argentinean Protected Areas with presence of Unionida

## Discussion

Understanding the distribution range of this group is critical to determining its state of decline. However, the group is often ignored or underestimated. In this regard, collection data can be used to determine this distributional range [50]. This study reports the results of an analysis of Argentina’s main malacological collections. It provides digital georeferenced data, essential for further studies on a regional scale, constituting a starting point for focusing conservation efforts on the taxa studied.

The more than 1,500 lots georeferenced in this work correspond to a broad time period (1900 to 2011). Thus, the maps created on the basis of this information correspond to known distribution limits of the organisms under analysis. Klunzinger et al. [40] report that such limits reflect the influence of environmental factors on distribution patterns of unionidan species. Factors such as change in land use, pollution, modifications in courses and entry of species with invasive potential, among others, may modify the distributional range presented. This approach provides a more accurate approximation to unionidan geographical patterns of distribution over the Surface Drainage Basins in the region. Furthermore, specific features of the naiad’s life cycle need to be considered [24], [25]. For example, some life cycles include a parasitic phase, some are very long-lived (certain European species live as long as 200 years) [48] and some individuals that reach maturity are potentially resistant to certain environmental changes, enabling them to survive for a long time [38], [39]. This particular life history is not necessarily shortened by environmental alterations such as the construction of dams and reservoirs [22]. Nevertheless, these alterations may displace fish populations and prevent them from taking part in unionidan species life cycles. In consequence, even though these populations persist today, they are liable to become extinct because they may be unable to complete their life cycle since their larvae would lack fish hosts to parasitize if the fish were extinct or displaced from the environment [50]. Thus, these populations have no future life expectation, being functionally extinct, as described by Parmalee and Bogan [51]. On a larger scale, this scenario can generate an *extinction debt* or *future extinction* of a great number of mussels caused by the habitat lost in the Neotropical Region [11], [52].

Previous studies in Argentina have focused on naiads’ distribution and developed maps based on the literature and analysis of collections [6], [9]. The present work also considered drainage basins and provided greater precision by using georeferenced information data in the distribution analysis. The naiad richness analysis in the Argentinean territory presented in this work tends to match the reference literature [6], [9], [17], [18], [20].

In South America, out of the 112 species cited by Pereira et al. [5], 20 are classified by the IUCN [53]. Of these, one species is *Critically Endangered* (*CR)*, two are *Vulnerable* (*VU*), and seventeen are considered *Not Threatened* categories (eleven are *Least Concern* (*LC*) and six are *Data Deficient* (*DD*)). Of the Unionida species cited for Argentina, only six (18%) have been classified by the IUCN [53], four of which are *Least Concern* (*Anodontites (A*.*) tenebricosus*, *A*. *(Lamproscapha) ensiformis*, *Diplodon (D*.*) chilensis* and *D*. *(D*.*) parallelopipedon*), while two are *Data Deficient* (*A*. *(A*.*) elongatus and D*. *(D*.*) hylaeus*). The neighboring countries have different percentages, and in all cases, the number of species with *Not Threatened* categories or *Not Evaluated* (NE) is greater (Table 3). Brazil has twelve classified species (15%), Uruguay has six (18%), Bolivia has eight (40%), Paraguay has six (22%) and Chile has one (*D*. *(D*.*) chilensis*), classified as *Least Concern* [53].

**Table 3:** IUCN categorization for the unionidan of Argentina and neighboring countries [53]. *Critically Endangered* (*CR)*, *Endangered (EN)*, *Vulnerable* (*VU*), *Near Threatened (NT)*, *Least Concern (LC)*, *Data Deficient (DD)*, *Not Evaluated (NE)*.

	CR	EN	VU	LC	DD	NE	Total of species
**Argentina**	0	0	3	5	4	70	82
**Brazil**	0	0	3	5	4	70	82
**Uruguay**	0	0	1	3	2	28	34
**Bolivia**	0	0	0	5	3	12	20
**Paraguay**	0	0	1	4	1	21	27
**Chile**	0	0	0	1	0	0	1

Regional efforts to classify the Unionida species in Brazil and Uruguay vary. Their results differ from the ones in the IUCN Red List [53]. In Brazil, 26 species (31%) were included in the Brazil Red Book [54] and in Uruguay, 30 species (88%) were considered *Species Prioritized for Conservation* [55]. However, a recent re-evaluation of the Brazil Red Book conducted by the Instituto Chico Mendes para a Conservação da Biodiversidade [56], with a rigid application of the IUCN criteria, listed only two species as threatened with the *Endangered* (EN) category, leaving 24 species excluded from the previous Brazil Red Book [54]. It seems unlikely that the environmental conditions of the freshwater environment of Brazil has improved over these few years [11], [57]. This leads to a problem related to the use of IUCN global criteria for regional analysis, especially in groups like the Neotropical Unionida where the lack of basic information is so important.

Spatial analysis showed that almost half of the basins had naiads present in at least one protected area. However, less than 14% of the APA contains records of species of unionidan and only 9% contains records of four or more species. This percentage clearly establishes how urgent it is to create new protected habitats for freshwater environments where the populations of naiads can grow and stabilize.

Despite being considered a hotspot of freshwater biodiversity, The Plata Basin is also the hydrographic system with least protected areas of Argentina and, according to the World Wildlife Fund, one the world’s ten most threatened river basins [58]. The major threats for this freshwater environment are habitat loss caused by dams and infrastructure projects, pollution, invasive species and climate change [58], [59], all of which affect freshwater bivalve populations directly [5]. All these threats are not independent, having a synergistic impact on freshwater habitats. For example, the construction of dams changes the dynamics of the river transforming it into a lentic environment and reducing the sediments flowing downstream [57]. These changes have allowed the settlement and colonization of invasive species as *C*. *fluminea* and *L*. *fortunei* in the high Paraná River in Brazil [5]. Another impact related to the construction of dams is the loss of host fishes and the interruption of the dispersion of parasitic larvae. The Plata Basin is also considered the hydrographic region with the highest richness and the greatest diversity of freshwater gastropods, with a high number of species and endemism [9], [46], [60]. These considerations are also valid for freshwater bivalves, with a high richness compared to others hydrographic systems of southern South America [5], [9], [61]. Despite this particular richness and high diversity of freshwater mollusks, to date there is no initiative, governmental or private, for the creation of protected areas for the conservation of this important group of animals. This shows that conservation management and a shift in the design of new protected areas are necessary for preserving the diversity of freshwater fauna in general and of Unionida in particular. The results of the present study will enable future research to enable the estimation of endangered status and degree of conservation of unionidan species such as by detecting priority areas for conservation, selecting of areas for population studies, and species modelling linking distributional ranges with environmental variables and climatic change.

## Supporting information

S1 Appendix Database of unionida specimens of the Argentina’s main malacological collections reviewed in the present work. (XLSX)
